# Associations of short-term changes in air pollution with spontaneous preterm birth: A time-stratified case-crossover analysis

**DOI:** 10.1016/j.annepidem.2026.110291

**Published:** 2026-09-17

**Authors:** Timothy D. Nelin, Nicolas P. Goldstein Novick, Joshua K. Radack, Rachel F. Ledyard, Allan C. Just, Sara B. DeMauro, Kristan A. Scott, Aimin Chen, Scott A. Lorch, Heather H. Burris

**Affiliations:** a Department of Pediatrics, University of Pennsylvania Perelman School of Medicine, Philadelphia, PA, USA; b Division of Neonatology, Children’s Hospital of Philadelphia, Philadelphia, PA, USA; c Philadelphia Regional Center for Children’s Environmental Health, Philadelphia, PA, USA; d Department of Epidemiology, Brown University, Providence, RI, USA; e Department of Biostatistics, Epidemiology, and Informatics, University of Pennsylvania Perelman School of Medicine, Philadelphia, PA, USA; f Center of Excellence in Environmental Toxicology, University of Pennsylvania Perelman School of Medicine, Philadelphia, PA, USA; g Department of Obstetrics and Gynecology, University of Pennsylvania Perelman School of Medicine, Philadelphia, PA, USA

**Keywords:** Preterm Birth, Spontaneous preterm birth, Air pollution, Fine particulate matter, Timestratified, Case-crossover

## Abstract

Spontaneous preterm birth (sPTB) is a leading cause of neonatal morbidity in the United States. Genetics incompletely explains sPTB risk and environmental contributions remain poorly understood. We analyzed associations of short-term fine particulate matter (PM_2.5_) exposure with sPTB in two datasets: 109,735 sPTBs in Michigan (2003–2020) and 5321 sPTBs in metropolitan Philadelphia (2008–2020). We used a time-stratified case-crossover design and daily high-resolution PM_2.5_ estimates to evaluate exposures in the week prior to sPTB using three complementary approaches: (1) nested daily lag terms; (2) averaged lag exposures; and (3) distributed lag non-linear models (DLNMs). To assess whether allowing flexible lag structures improved model fit, we performed likelihood ratio tests comparing models with linear lag terms to DLNMs. All models were adjusted for temperature. Across both cohorts and modeling approaches, we did not detect statistically significant associations of short-term PM_2.5_ exposure with sPTB, with point estimates close to the null with narrow confidence intervals. These findings suggest that, at the studied concentrations, short-term elevations in PM_2.5_ are unlikely to be associated with sPTB. As environmental regulatory protections evolve, pollution levels may change, underscoring the importance of continued surveillance of PM_2.5_-related health effects.

## Introduction

Preterm birth (PTB), defined as birth before 37 completed weeks of gestation, remains a leading cause of neonatal morbidity and mortality in the United States, but is not a uniform entity [1,2]. Spontaneous PTB (sPTB), resulting from preterm labor or preterm premature rupture of membranes, accounts for approximately 60% of PTB cases and differs clinically and biologically from medically indicated PTB (mPTB), which occurs via induction of labor or cesarean birth in the context of maternal or fetal complications such as preeclampsia or fetal growth restriction [3–7]. PTB is frequently studied as a single outcome, which may mask associations that are specific to the sPTB or mPTB [8].

Exposure to ambient fine particulate matter less than 2.5 microns in diameter (PM_2.5_), a major component of air pollution, has been associated with PTB [9–13]. While the more commonly studied long-term (e.g., whole pregnancy, trimester averages) PM_2.5_ exposures may increase PTB risk through chronic inflammatory and vascular mechanisms and are most often studied, it is also possible that short-term elevations in PM_2.5_ levels in the days preceding birth could trigger acute pathophysiologic responses that precipitate spontaneous labor or rupture of membranes.

We sought to address these gaps in the current literature by evaluating the association of granular, short-term PM_2.5_ exposure with sPTB using a time-stratified case-crossover design in two large, geocoded birth cohorts. The first was a population-based cohort from Michigan and the second was a hospital system-based in metropolitan Philadelphia, *GeoBirth*. This dual-cohort approach allowed for examination of variation by geographic context and data structure, as well as validation of findings. We hypothesized that short-term elevations in PM_2.5_ exposure in the week preceding birth would be associated with sPTB.

## Methods

### Study populations

The first retrospective cohort was composed of statewide Michigan birth records from January 1, 2003 through December 31, 2020, created by linking Michigan Department of Health birth certificates and infant death certificates up to a year after birth using name and date of birth. These files were probabilistically matched with maternal and newborn hospital discharge records using hospital, date of birth, residential ZIP code, insurance status, birth weight, gestational age, and race/ethnicity with previously established methods, achieving a 96% match rate [14]. Analysis was limited to liveborn, singleton, sPTBs with a gestational age of 20 to < 37 weeks and birthweight of no more than 5 standard deviations away from the mean birth weight for a given gestational age, as these discrepancies often indicate coding errors in either the birth weight or gestational age variable [15]. The second retrospective cohort of births was derived from the *GeoBirth* study of metropolitan Philadelphia, a curated dataset of manually chart-reviewed birth records from all singleton births at two urban academic hospitals in Philadelphia from January 1, 2008 through December 31, 2020 [16].

### Exposure

PM_2.5_ estimates were generated using XGBoost-IDW Synthesis (XIS), a daily high-resolution PM_2.5_ machine-learning model covering the contiguous US from 2003 to 2024 [17]. XIS uses satellite aerosol optic depth and a parsimonious set of satellite, land-use, meteorological, and topographical factors to make estimates at arbitrary points, capturing near-roadway gradients allowing approximation of local PM_2.5_ exposures with greater precision [17,18]. Daily temperature predictions were generated by XIS-temperature, which is also based on a machine-learning model accounting for land-use and vegetation among other factors [17,19].

The Michigan dataset has maternal residential census tract for each birth, which is used for Geographic Information System (GIS) linkage. For *GeoBirth*, maternal residential addresses for each birth were geocoded using ArcMAP Version 10.8 and ArcGIS Street Map Premium North America 2021.1 address locator, using a minimum match score of 75 and assigning census block group based on the 2010 U.S. Census boundaries [16]. For this analysis, daily exposures were assigned at the respective census unit’s center of population.

### Outcome

The primary outcome was sPTB. For the Michigan cohort, we used maternal and newborn linked records to define sPTB based on an algorithm utilizing birth certificate and ICD-9/10 codes to identify premature rupture of membranes, premature or preterm labor, or tocolysis at a gestational age of < 37 weeks (Supplemental Figure 1). For the *GeoBirth* cohort, two independent research assistants used a standardized protocol to characterize the primary reason for birth prior to 37 weeks’ gestation [16,20]. PTBs that resulted from preterm labor or spontaneous rupture of membranes were deemed spontaneous.

### Statistical analysis

To estimate the association of short-term elevations in PM_2.5_ exposure with sPTB, we used a time-stratified case-crossover design. This approach inherently controls for time-invariant confounders by comparing each subject’s exposure on the event day (i.e., date of sPTB) to their own exposure on referent (control) days within the same temporal stratum [21–23]. We used a time-stratified referent selection approach, matching control days on the same day of the week, within the same month and year, to account for confounding by day-of-week effects, long-term time trends, and seasonal variation [23]. We used three complementary modeling approaches within the case-crossover framework to examine associations of short-term elevations in PM_2.5_ exposure with sPTB, per 5 μg/m^3^ of PM_2.5_. All lag models were fit using a conditional logistic regression framework (clogit function in R), stratifying on individual to ensure that each subject serves as their own control [24]. First, we fit a single model with mutually adjusted terms for daily PM_2.5_ exposures on lag days 0 through 6, allowing evaluation of day-specific effects while jointly adjusting for correlated exposures across time [24]. We selected lags 0–6 a priori to capture potential acute inflammatory or vasoactive triggering during the days immediately preceding delivery while limiting collinearity across correlated daily exposures [25,26]. Second, we derived cumulative PM_2.5_ exposures by averaging PM_2.5_ across selected lag windows in the week prior to each sPTB, evaluating windows from 1 through 7 days. These moving averages reduce collinearity across individual lag terms, improve model stability, and enhance interpretability by yielding a single effect estimate per window [27,28]. Third, we fit time-stratified case-crossover models including distributed lag non-linear terms to examine the temporal pattern of risk across daily PM_2.5_ exposures on the day of the outcome (lag 0) through the 6 days prior (lags 1–6) using the dlnm package in R [29,30]. We constructed cross-basis functions to simultaneously model the exposure-response and lag-response relationships over lags 0 through 6 days. For each exposure, the exposure-response function was specified using a natural cubic spline with 3 ° of freedom, allowing for potential non-linear relationships between exposure level and outcome. The lag-response function was similarly specified using a natural cubic spline with 3 ° of freedom, allowing for flexible, non-linear lag structures across the 7-day exposure window. Predicted associations across exposure levels and lags were obtained using standard DLNM prediction functions, with effects centered at the mean exposure level. To assess whether the distributed lag non-linear model (DLNM) improved model fit, we performed an omnibus likelihood ratio test comparing models including the PM_2.5_ cross-basis term to models including linear PM_2.5_ terms across the same lag window.

To account for potential confounding by ambient temperature, averaged and daily lag models included linear and quadratic terms for mean daily temperature, while distributed lag models included a temperature cross-basis function.

We performed *post-hoc* analyses to assess potential heterogeneity and the robustness of our findings. We stratified by urbanicity in Michigan and insurance status in both cohorts to evaluate variation across geographic and socioeconomic contexts. To assess sensitivity to differences in spatial resolution between cohorts, we repeated the GeoBirth analyses after averaging block group exposures to the census tract. To evaluate potential residual confounding by extreme heat, we replaced the linear and quadratic temperature terms with an indicator for daily mean temperature exceeding the cohort-specific 90th percentile of summer temperatures. Finally, to assess sensitivity to outcome classification, we repeated the primary analyses using any PTB as the outcome.

The local Institutional Review Board deemed this study non-human subjects research per 45 CFR 46.104 for the Michigan state dataset and approved the use of the GeoBirth dataset (IRB# 829674). All analyses were performed using R statistical software (version 4.5.2; R Core Team 2025).

## Results

In Michigan, we identified 109,735 eligible instances of sPTB (6% of total, 73% of PTB) (Supplemental Figure 2). The median gestational age was 35 weeks (IQR 33–36). The median birthweight was 2.4 kg (IQR 1.9–2.8) (Table 1). Mean (SD) PM_2.5_ exposure in the 7-days prior to birth for the study population was 10.2 (4.1) μg/m^3^. In *GeoBirth*, we identified 5321 eligible instances of sPTB (6% of total, 50% of PTB) (Supplemental Figure 2). The median gestational age was 35 weeks (IQR 31–36). Median birthweight was 2.3 kg (IQR 1.5–2.7) (Table 1). Mean (SD) PM_2.5_ exposure for the study population in the 7-days prior to birth was 10.1 (3.5) μg/m^3^. Daily average PM_2.5_ concentrations decreased across Michigan and metropolitan Philadelphia during the study period (Fig. 1). Fig. 2 displays the geographic distribution of births for the Michigan (2A) cohort and *GeoBirth* (2B) cohort in metropolitan Philadelphia over the study period. Geographic variation in concentration of births for *GeoBirth* represents both population density and the health system’s catchment. Fig. 3 displays geographic variation in PM_2.5_ exposure across four Tuesdays in July of 2015.

We found no statistically significant evidence of an association of PM_2.5_ (per 5 μg/m^3^) with sPTB in any model. For the daily nested lag models, odds ratios (95% CI) ranged from 0.993 (0.985, 1.001) to 1.006 (0.998, 1.014) in Michigan and from 0.967 (0.932, 1.004) to 1.042 (0.999, 1.086) in *GeoBirth*. Odds ratios (95% CI) for averaged lag models ranged from 0.991 (0.980, 1.001) to 0.999 (0.991, 1.006) in Michigan and from 0.984 (0.952, 1.017) to 1.013 (0.962, 1.067) in *GeoBirth* (Table 2).

The DLNM provided modest evidence of improved model fit in Michigan (omnibus likelihood ratio test *p* = 0.05) but not in GeoBirth (*p* = 0.16). Fig. 4 displays the DLNM-derived lag-response associations for mean PM_2.5_ exposure. Effect estimates were small, with no clear evidence of a significant association across lags.

Associations of short-term PM_2.5_ exposure with sPTB were largely consistent across strata of urbanicity in the Michigan cohort, with no consistent evidence of heterogeneity across metropolitan, micropolitan, or non-core counties (averaged lag 0–6 ORs, 0.985–0.991; Supplemental Table 1). Stratified analyses by insurance status also demonstrated similar associations across groups in both cohorts (Michigan ORs, 0.976–1.002; GeoBirth ORs, 1.014–1.023; Supplemental Tables 2 and 3). Findings were materially unchanged after tract-level aggregation in *GeoBirth*, alternative adjustment for extreme heat, or use of any PTB as the outcome (Supplemental Tables 4–6).

## Discussion

In two distinct datasets, we detected no association of short-term PM_2.5_ with sPTB. The Michigan dataset was a large, state-wide, population-based dataset spanning 18 years with PM_2.5_ exposure at the census tract level and sPTB phenotyping determined by administrative codes. The *GeoBirth* dataset based in metropolitan Philadelphia was a smaller, health system-based dataset spanning 13 years, with PM_2.5_ exposures at the census block group level (smaller than census tract), with rigorously phenotyped sPTBs based on clinical documentation. Results were also substantively identical across multiple modeling strategies using nested daily lags, averaged lags, and DLNM methods. These null findings suggest that short-term elevations in PM_2.5_, in the observed matched exposure range are unlikely to trigger sPTB.

These findings add to a literature with variable results across region, exposure level, exposure timing, severity of PTB, PTB phenotype, and study design [8]. While most prior research utilizes pregnancy averaged or trimester-specific averaged PM_2.5_ exposures, our results are consistent with several of these studies. A study that partially overlaps with our sample found no association of PM_2.5_ exposure over the entire pregnancy with PTB among a cohort of 7961 births in Detroit, MI from 2008 to 2010 [31]. Similarly, a retrospective cohort study by Johnson et al. evaluated the association of first and second trimester PM_2.5_ exposure with sPTB in a cohort of 258,294 births in New York City and did not find a significant association [32]. Conversely, other retrospective cohort studies have reported positive associations, including three studies from Shanghai, China finding risks associated with second or third trimester exposures [33–35]. In a study of 63,027 births from Beersheba, Israel (2003–2013), researchers found a positive association of PM_2.5_ with sPTB amongst Bedouin women specifically on lag day 0 [36]. In contrast, a study of 1318,570 births from New South Wales, Australia (2001–2019) looking at exposure in the week before birth found that PM_2.5_ exposure was protective against sPTB [37]. With regard to the potential for a non-linear exposure-response relationship, the DLNM provided modest evidence of improved model fit in Michigan, although the corresponding lag-response curve did not show a clear or biologically coherent increase in risk concentrated at a particular lag, and the finding was not replicated in *GeoBirth*. We therefore view this as hypothesis-generating. Nonlinear lag structures may merit evaluation in populations with broader exposure distributions rather than evidence of a delayed or nonlinear association in our data.

One explanation for the heterogeneity in the literature is geographic variation in PM_2.5_ concentrations and composition. The studies with non-significant findings from New York City, NY and Detroit, MI had median PM_2.5_ levels around 11 and 12 μg/m^3^, respectively, which are similar to the levels observed in Michigan and Philadelphia [31,32]. The three studies using birth data from Shanghai, China that found significant associations of PM_2.5_ with sPTB risk had median PM_2.5_ levels of 53, 43, and 49 μg/m^3^ [33–35]. The median PM_2.5_ levels in the study from Israel that found higher risk with short-term PM_2.5_ exposure were around 20 μg/m^3^ in the summer and 22 μg/m^3^ in the winter [36]. Amongst the studies conducted in Australia that found protective effects, the median PM_2.5_ levels were low, both around 7 μg/m^3^ [37,38]. Additionally, geographic variation in PM_2.5_ composition may contribute to heterogeneity in observed associations, as differing chemical constituents may have distinct biological effects [39].

Importantly, we found that average annual PM_2.5_ concentrations decreased over the study period. We interpret this as demonstrable success of the Clean Air Act, in addition to state and local policy, aimed at reducing fine particulate matter exposure [40–42]. Thus, it is important to interpret our null findings through the lens of the substantial reduction in annual average PM_2.5_ exposure. If the Clean Air Act were to be altered to allow for a higher daily or annual average PM_2.5_ exposure, our findings may no longer be representative of the association of short-term fine particulate matter exposure and sPTB in Michigan and metropolitan Philadelphia.

The lack of clear mechanisms underlying sPTB pathogenesis also contributes to poor understanding. Hypothesized mechanisms include localized inflammatory responses as many, but not all, placentas from sPTBs show signs of either acute or chronic inflammation [43]; vascular dysfunction contributing to placental insufficiency [44]; and disruptions of hormonal signaling cascades, such as the hypothalamic-pituitary-adrenal axis, that may trigger early parturition [45]. These processes likely interact, further complicating the underlying process. Given the inflammation and oxidative stress induced by PM_2.5_, it remains plausible that PM_2.5_ may contribute to sPTB risk at higher levels and longer exposure durations, but future work and ongoing surveillance are needed, especially if PM_2.5_ levels rise [25,46].

Our study has several strengths, including the use of two large, complementary birth cohorts with detailed geospatial exposure data and rigorous outcome phenotyping. The Michigan cohort was a population-based cohort and *GeoBirth* used detailed, duplicate, manual chart reviews for sPTB phenotyping. The XIS model is a state-of-the-art, granular PM_2.5_ exposure model that outperforms most others [17]. Specifically, the XIS model has a 17% reduction in the weighted mean absolute error of predictions and stronger relationship between PM_2.5_ and social vulnerability compared to the Fused Air Quality Surface Using Downscaling product from the United States Environmental Protection Agency, supporting its application in modeling environmental exposures and human health outcomes [17]. Notably, XIS-based PM_2.5_ exposure estimates have been associated with adverse birth outcomes, including reduced birth weight, in large, independent cohorts, supporting the validity of this exposure modeling approach [47]. The use of three modeling strategies that all found the same results across two cohorts is also a strength, and not commonly done for studies of PM_2.5_ association with PTB.

Our study has limitations. PM_2.5_ exposure was assigned using residence at birth and did not account for indoor exposures, air filtration, or time spent away from home. Exposure assignment was less spatially granular in Michigan than in *GeoBirth*, although tract-level aggregation of *GeoBirth* estimates produced nearly identical findings. The Michigan sPTB definition relied on administrative data and may have misclassified some medically indicated births as spontaneous. Results were similarly null in the manually adjudicated *GeoBirth* cohort and when any PTB was used as the outcome, although these analyses cannot eliminate potential outcome misclassification. Findings may not generalize beyond the two regions and exposure distributions studied. Our findings may also be influenced by selection bias, as case-crossover analyses of live births condition on fetuses that survive to delivery. If short-term PM_2.5_ exposure increases the risk of stillbirth, this could attenuate observed associations with preterm birth among surviving pregnancies. Several co-occurring factors that track with air pollution could modify responses to short-term elevations in air pollution. For example, individuals living in areas of higher baseline levels of pollution could also be more likely to be exposed to tobacco or poverty. At the neighborhood level, PM_2.5_ exposure remains spatially patterned, disproportionately affecting communities of color and neighborhoods with higher levels of socioeconomic disadvantage [48,49]. It is possible that short term elevations from higher or lower baselines could have differential effects, serving as effect modifiers. We were reassured that rural/urban stratification and by insurance status did not meaningfully change results. Nonetheless, we did not test for every potential effect modifier. Lastly, we lacked spatially and temporally resolved co-pollutant estimates linked to individual births, and residual confounding by NO_2_, ozone, or other time-varying exposures remains possible.

In conclusion, we detected no associations of short term PM_2.5_ with sPTB in two cohorts using multiple modeling techniques. These null results are informative, as they were nearly identical in two distinct cohorts. As such, at current exposure levels in Michigan and metropolitan Philadelphia, acute PM_2.5_ exposure alone is unlikely to be a catalyst for sPTB. We speculate these results are indicative of the success of environmental policies that have reduced PM_2.5_ to concentrations not associated with this outcome in these populations and recommend that if levels rise with loosening of pollution regulations, ongoing surveillance of effects of PM_2.5_ should remain a public health priority.

## Supplementary Material

Supplement

Supplementary data associated with this article can be found in the online version at doi:10.1016/j.annepidem.2026.110291.

## Figures and Tables

**Fig. 1.**
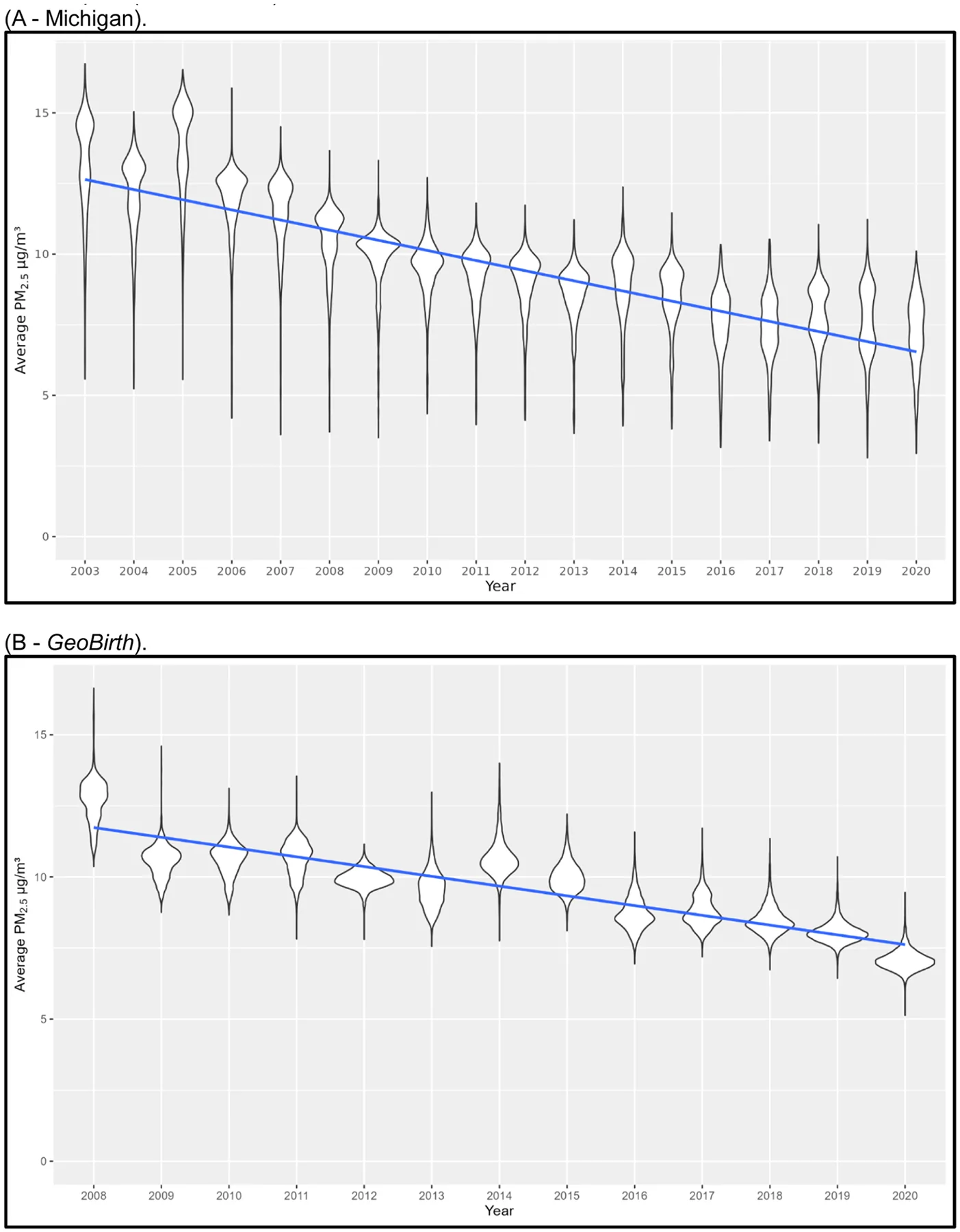
Decreases in annual average PM_2.5_ in Michigan (A, 2003–2020) and metropolitan Philadelphia (B, 2008–2020) over time. (A - Michigan). Abbreviations: PM_2.5_, fine particulate matter < 2.5 microns in diameter. Annual PM_2.5_ levels are displayed with violin plots, which represent the distribution of kernel density estimates to indicate PM_2.5_ density and variability. The blue lines represent fitted linear trends in PM_2.5_ over time.

**Fig. 2.**
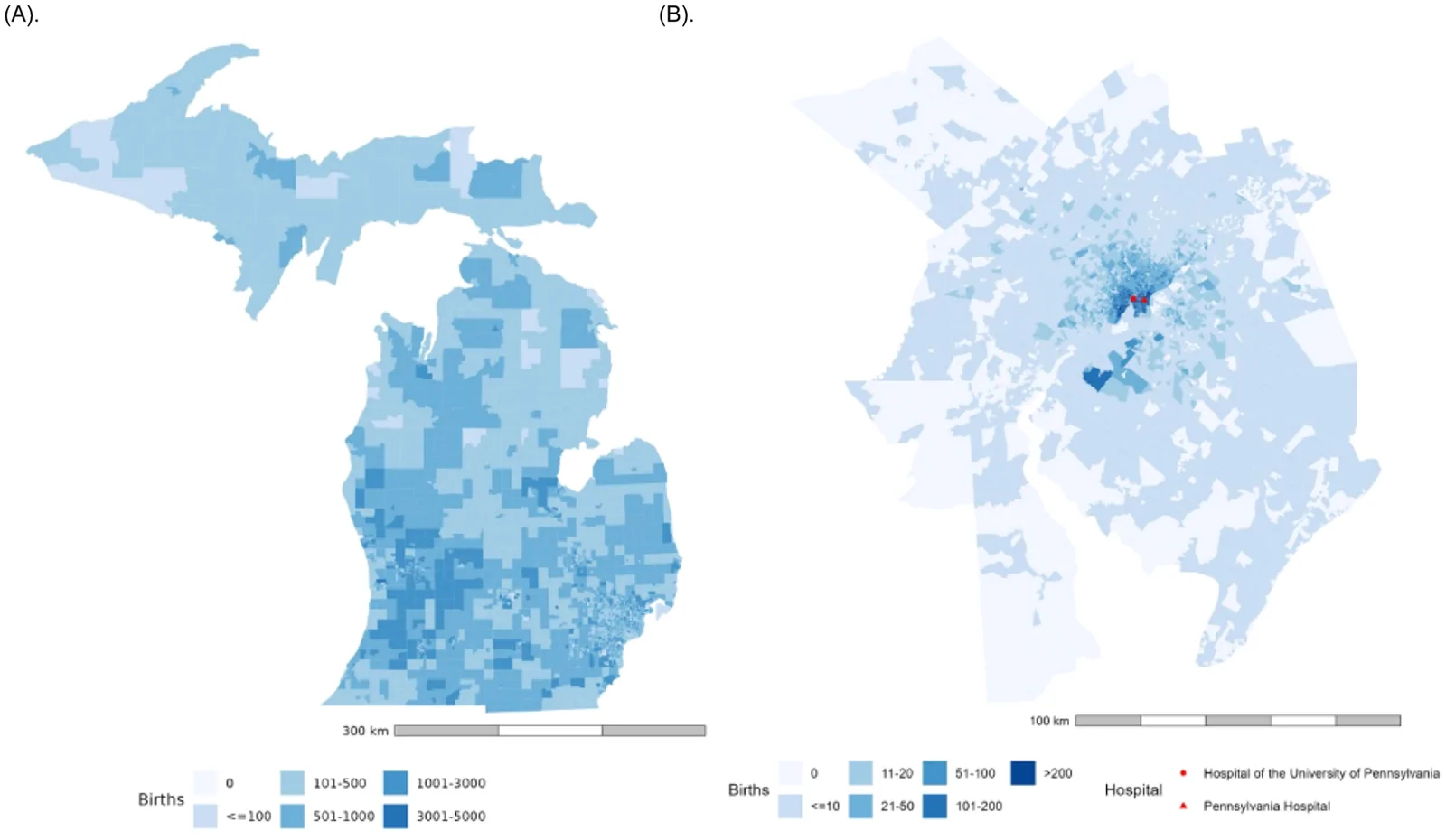
Geographic variation in birth volume in Michigan (left, A) and *GeoBirth in* metropolitan Philadelphia (right, B). Figure depicts the census tract at time of birth for the Michigan birth cohort and the census block group at time of birth for the *GeoBirth* cohort in metropolitan Philadelphia over the study period highlighting geographic variation in birth volume in Michigan and metropolitan Philadelphia.

**Fig. 3.**
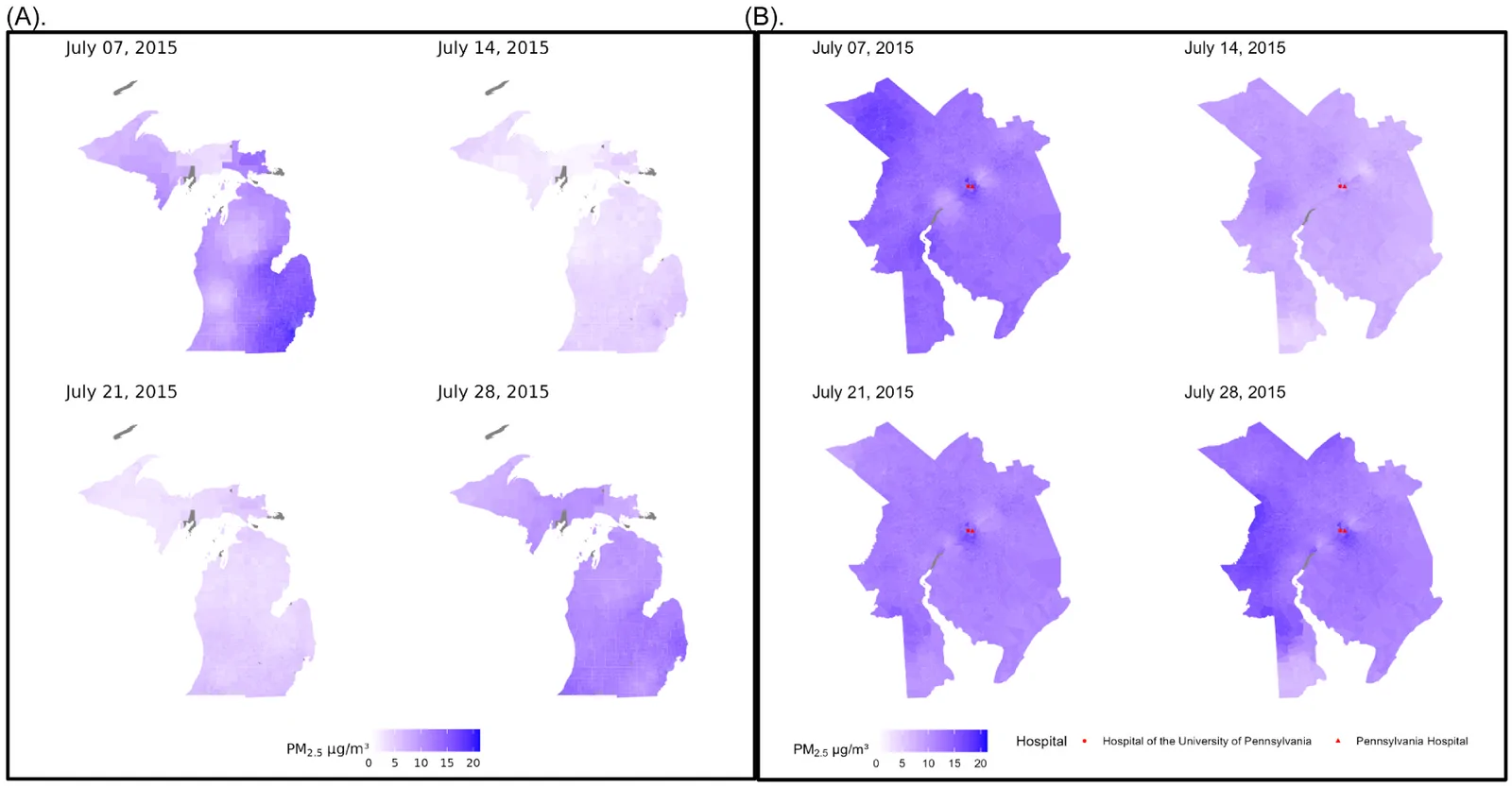
Example variation of the PM_2.5_ concentrations on the four Tuesdays in July 2015 in Michigan (left, A) and metropolitan Philadelphia (right, B). Abbreviations: PM_2.5_, fine particulate matter < 2.5 microns in diameter. In the Michigan dataset, PM_2.5_ annual levels were assigned at the census tract level based on Geographic Information System linkage. In the *GeoBirth* dataset in metropolitan Philadelphia, PM_2.5_ annual levels were assigned at the census block group level based on geocoded residential address. PM_2.5_ level is highlighted by purple shading at the census tract level for Michigan and at the census block group level for *GeoBirth*. Gray shading indicates no PM_2.5_ exposure data was available in that region. The locations of the hospitals in *GeoBirth* are highlighted in red.

**Fig. 4.**
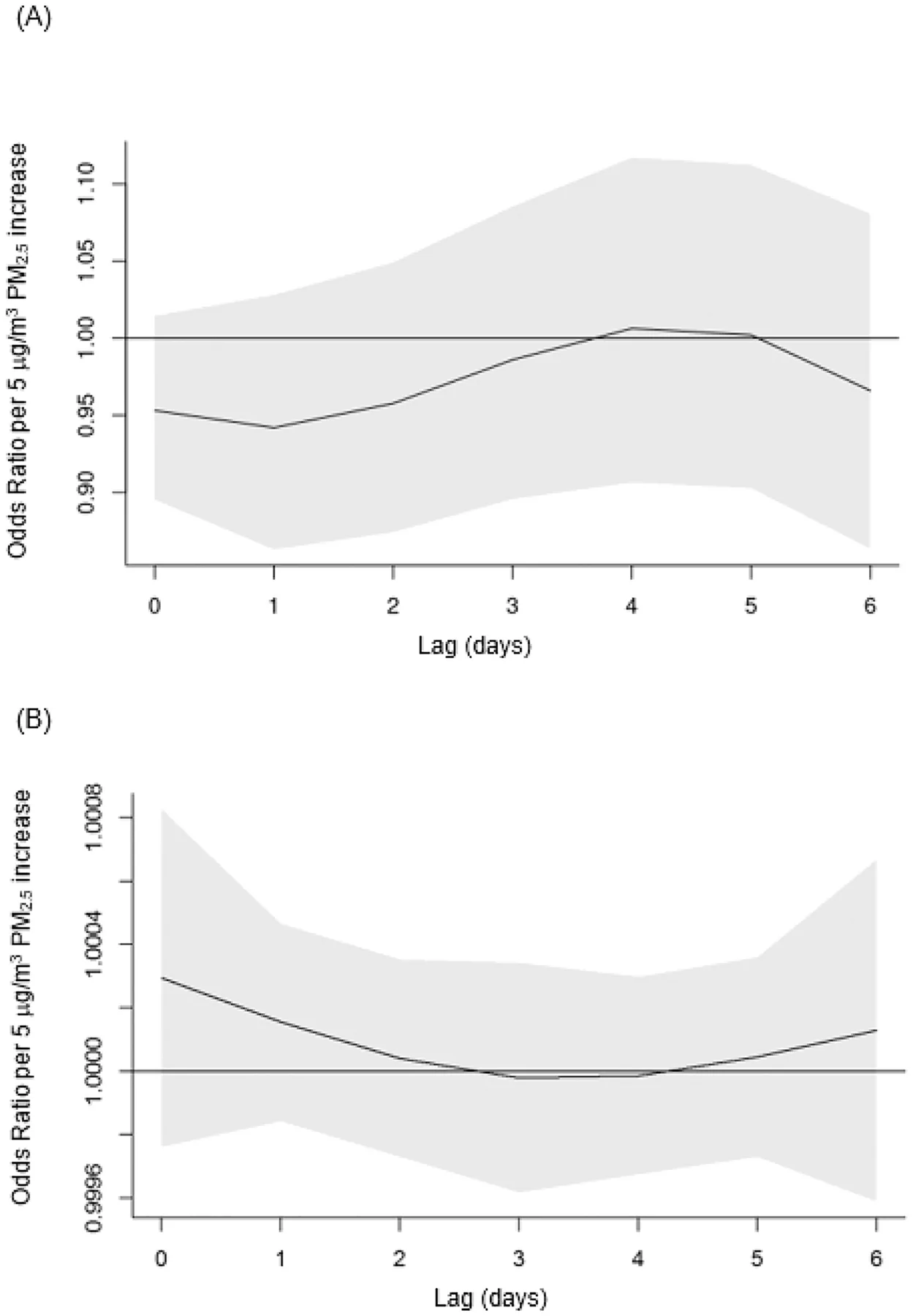
Lag-response associations of short-term PM_2.5_ exposure with spontaneous preterm birth estimated using distributed lag non-linear models in the Michigan cohort (A) and *GeoBirth* Cohort (B). Abbreviations: sPTB, spontaneous preterm birth; DLNM, distributed lag non-linear model; PM_2.5_, particulate matter < 2.5 μm in diameter. Lag association showing estimated odds ratios for sPTB over lags 0–6 days. Estimates were derived from DLNMs within a time-stratified case-crossover framework, and expressed as relative odds for a 5 μg/m^3^ increase in PM_2.5_ evaluated at the cohort mean (Michigan, 10.2 μg/m^3^; *Geobirth*, 10.1 μg/m^3^). Shaded areas represent 95% confidence intervals.

**Table 1:** Characteristics of singleton spontaneous preterm births (sPTB) in the Michigan dataset from 2003 to 2020 and the *GeoBirth* dataset with a geocoded address in metropolitan Philadelphia, from 2008 to 2020.

Characteristic	Michigan (n = 109,735)	*GeoBirth* (n = 5321)
	n (column %)	n (column %)
Gestational Age (weeks), Median (IQR)	35 (33, 36)	35 (31, 36)
Birthweight (kilograms), Median (IQR)	2.4 (1.9, 2.8)	2.3 (1.5, 2.7)
Maternal Age (years)		
< 25	37,605 (34)	1801 (35)
25–34	55,610 (51)	2578 (50)
≥ 35	16,520 (15)	826 (16)
Married	35,599 (48)	1490 (28)
Race/Ethnicity		
Non-Hispanic Black	31,062 (28)	3542 (67)
Non-Hispanic White	65,579 (60)	923 (18)
Hispanic	6972 (6)	369 (7)
Asian/Pacific Islander	3317 (3)	248 (5)
Multiracial/Other	2495 (2)	138 (3)
Unknown	310 (0.3)	38 (1)
Insurance Status		
Public	52,048 (47)	2587 (60)
Private	51,183 (47)	1721 (40)
Self-Pay/Other	6504 (6)	35 (1)
Pre-Pregnancy BMI (kg/m^2^), Median (IQR)	25 (22, 31)	25 (22, 31)
Nulliparous	30,857 (28)	2092 (39)
Pre-Existing Hypertension	2841 (3)	429 (8)
Hypertensive Disorder of Pregnancy	9228 (9)	555 (10)
Severe HTN/Pre-Eclampsia/Eclampsia/HELLP*	7649 (7)	261 (5)
Any Diabetes	7855 (7)	294 (6)
Any Smoking	24,021 (22)	1565 (33)
Year		
2003–2007	36,798 (34)	–
2008–2011	26,000 (24)	1923 (36)
2012–2015	21,739 (20)	1620 (30)
2016–2020	25,198 (23)	1778 (33)
7 Days Pre-Birth Daily Mean Temperature (Celsius), mean (SD)	9.8 (10.1)	13.7 (9.3)
Pregnancy Daily Mean PM_2.5_ (μg/m3), mean (SD)	10.2 (2.4)	10.1 (1.8)
7 Days Pre-Birth Daily Mean PM_2.5_ (μg/m3), mean (SD)	10.2 (4.1)	10.1 (3.5)

**Abbreviations:** BMI, body mass index; GA, gestational age; HTN, hypertension; HELLP, hypertension, elevated liver enzymes, low platelets; PM_2.5_, particulate matter < 2.5 μm/m^3^.

* For Geobirth, limited to Pre-Eclampsia, Eclampsia, and/or HELLP.

**Michigan Missing Data:** Birthweight (n = 7), Married (n = 35,401), Pre-Pregnancy BMI (not reported before 2007; missing data 2007–2020 n = 11,242 or 14%), Nulliparous (n = 395), Pre-Existing Hypertension (n = 714), Hypertensive Disorder of Pregnancy (n = 714), Any Diabetes (n = 714), Any Smoking (n = 1177).

***GeoBirth* Missing Data:** Gestational Age Days (n = 53), Birthweight (n = 5), Maternal Age (n = 116), Married (n = 47), Race/Ethnicity (n = 63), Insurance Status (n = 978), Pre-Pregnancy BMI (n = 1774), Pre-Existing Hypertension (n = 25), Hypertensive Disorder of Pregnancy (n = 25), Any Diabetes (n = 563), Any Smoking (n = 563).

**Table 2:** Associations of PM_2.5_ with spontaneous preterm birth (sPTB) using daily nested lag and averaged lagged time-stratified case-crossover models in two birth cohorts, Michigan (n = 109,735) and *GeoBirth* in Metropolitan Philadelphia (n = 5321).

Michigan (2003–2020)			
Daily Lag	sPTB OR (95% CI)	Averaged Lag	sPTB OR (95% CI)
Lag 0	0.994 (0.987, 1.001)	Lag 0	0.997 (0.994, 1.000)
Lag 1	1.006 (0.998, 1.014)	Lag 0–1	0.999 (0.991, 1.006)
Lag 2	0.995 (0.988, 1.003)	Lag 0–2	0.998 (0.991, 1.005)
Lag 3	1.004 (0.992, 1.017)	Lag 0–3	0.998 (0.990, 1.007)
Lag 4	0.999 (0.991, 1.008)	Lag 0–4	0.997 (0.986, 1.007)
Lag 5	0.993 (0.985, 1.001)	Lag 0–5	0.993 (0.982, 1.003)
Lag 6	0.999 (0.992, 1.006)	Lag 0–6	0.991 (0.980, 1.001)
*GeoBirth* (2008–2020)			
Daily Lag	sPTB OR (95% CI)	Averaged Lag	sPTB OR (95% CI)
Lag 0	0.967 (0.932, 1.004)	Lag 0	0.984 (0.952, 1.017)
Lag 1	1.042 (0.999, 1.086)	Lag 0–1	1.003 (0.964, 1.042)
Lag 2	0.979 (0.939, 1.021)	Lag 0–2	1.004 (0.961, 1.048)
Lag 3	1.016 (0.975, 1.058)	Lag 0–3	1.010 (0.963, 1.060)
Lag 4	1.007 (0.967, 1.049)	Lag 0–4	1.013 (0.962, 1.067)
Lag 5	0.986 (0.946, 1.027)	Lag 0–5	1.008 (0.953, 1.065)
Lag 6	0.999 (0.964, 1.035)	Lag 0–6	1.005 (0.946, 1.066)

**Abbreviations:** OR = odds ratio, CI = confidence interval, sPTB = spontaneous preterm birth

Conditional logistic regression controlling for mean temperature + mean temperature squared, estimates presented per 5 μg/m^3^ of PM_2.5_.
