# Supplementary material for: Associations of short-term changes in air pollution with spontaneous preterm birth: A time-stratified case-crossover analysis
Source: Ann Epidemiol. Author manuscript; Available in PMC 2026 Oct 14. (PMC13662903; doi:10.1016/j.annepidem.2026.110291)
Supplement: Supplementary file 1 [file NIHMS2214102-supplement-Supplement.docx]

| **Supplemental Table 1.** Associations of PM_2.5_ with sPTB stratified by urbanicity, Michigan (2003-2020). | | | | |
| --- | --- | --- | --- | --- |
|  | **Daily Lag** | **sPTB OR (95% CI)** | **Averaged Lag** | **sPTB OR (95% CI)** |
| Metropolitan  (n = 95,865) | Lag 0 | 0.994 (0.987, 1.002) | Lag 0 | 0.994 (0.987, 1.002) |
|  | Lag 1 | 1.007 (0.999, 1.016) | Lag 0-1 | 1.000 (0.992, 1.008) |
|  | Lag 2 | 0.995 (0.986, 1.004) | Lag 0-2 | 0.999 (0.990, 1.008) |
|  | Lag 3 | 1.002 (0.993, 1.011) | Lag 0-3 | 0.999 (0.989, 1.008) |
|  | Lag 4 | 1.000 (0.991, 1.009) | Lag 0-4 | 0.997 (0.987, 1.008) |
|  | Lag 5 | 0.993 (0.984, 1.002) | Lag 0-5 | 0.993 (0.982, 1.004) |
|  | Lag 6 | 0.999 (0.992, 1.007) | Lag 0-6 | 0.991 (0.979, 1.002) |
| Micropolitan  (n = 8,766) | Lag 0 | 0.979 (0.949, 1.010) | Lag 0 | 0.979 (0.949, 1.010) |
|  | Lag 1 | 1.002 (0.966, 1.039) | Lag 0-1 | 0.982 (0.951, 1.014) |
|  | Lag 2 | 0.990 (0.955, 1.027) | Lag 0-2 | 0.987 (0.953, 1.022) |
|  | Lag 3 | 1.031 (0.994, 1.070) | Lag 0-3 | 0.996 (0.960, 1.034) |
|  | Lag 4 | 0.979 (0.944, 1.016) | Lag 0-4 | 0.991 (0.952, 1.031) |
|  | Lag 5 | 0.989 (0.955, 1.025) | Lag 0-5 | 0.986 (0.944, 1.030) |
|  | Lag 6 | 1.008 (0.979, 1.039) | Lag 0-6 | 0.988 (0.943, 1.035) |
| Non-core  (n = 4,994) | Lag 0 | 1.003 (0.960, 1.048) | Lag 0 | 1.003 (0.960, 1.048) |
|  | Lag 1 | 0.967 (0.921, 1.016) | Lag 0-1 | 0.978 (0.936, 1.021) |
|  | Lag 2 | 1.011 (0.962, 1.062) | Lag 0-2 | 0.984 (0.938, 1.032) |
|  | Lag 3 | 1.005 (0.957, 1.056) | Lag 0-3 | 0.995 (0.945, 1.047) |
|  | Lag 4 | 1.019 (0.970, 1.070) | Lag 0-4 | 1.004 (0.950, 1.060) |
|  | Lag 5 | 0.996 (0.950, 1.045) | Lag 0-5 | 1.000 (0.943, 1.061) |
|  | Lag 6 | 0.969 (0.930, 1.010) | Lag 0-6 | 0.985 (0.923, 1.051) |

**Abbreviations:** OR, odds ratio; CI, confidence interval; sPTB, spontaneous preterm birth.
Conditional logistic regression models using a time-stratified case-crossover design. Models adjusted for mean temperature and mean temperature squared. Estimates are presented per 5 µg/m^3^ increase in PM_2.5_.

| **Supplemental Table 2.** Associations of PM_2.5_ with sPTB stratified by insurance type, Michigan (2003-2020). | | | | |
| --- | --- | --- | --- | --- |
|  | **Daily Lag** | **sPTB OR (95% CI)** | **Averaged Lag** | **sPTB OR (95% CI)** |
| Private Insurance  (n = 51,136) | Lag 0 | 0.994 (0.984, 1.005) | Lag 0 | 0.994 (0.984, 1.005) |
|  | Lag 1 | 1.004 (0.992, 1.017) | Lag 0-1 | 0.998 (0.987, 1.009) |
|  | Lag 2 | 0.994 (0.982, 1.007) | Lag 0-2 | 0.995 (0.983, 1.007) |
|  | Lag 3 | 0.999 (0.987, 1.011) | Lag 0-3 | 0.993 (0.980, 1.006) |
|  | Lag 4 | 1.000 (0.988, 1.013) | Lag 0-4 | 0.989 (0.975, 1.003) |
|  | Lag 5 | 0.990 (0.978, 1.002) | Lag 0-5 | 0.982 (0.967, 0.997) |
|  | Lag 6 | 0.993 (0.983, 1.003) | Lag 0-6 | 0.976 (0.960, 0.992) |
| Public Insurance  (n = 52,008) | Lag 0 | 0.991 (0.980, 1.002) | Lag 0 | 0.991 (0.980, 1.002) |
|  | Lag 1 | 1.011 (0.999, 1.024) | Lag 0-1 | 0.999 (0.988, 1.010) |
|  | Lag 2 | 0.990 (0.978, 1.002) | Lag 0-2 | 0.999 (0.987, 1.012) |
|  | Lag 3 | 1.012 (0.999, 1.025) | Lag 0-3 | 1.002 (0.988, 1.015) |
|  | Lag 4 | 0.996 (0.984, 1.009) | Lag 0-4 | 1.002 (0.987, 1.017) |
|  | Lag 5 | 0.996 (0.984, 1.009) | Lag 0-5 | 1.001 (0.985, 1.018) |
|  | Lag 6 | 1.005 (0.995, 1.016) | Lag 0-6 | 1.002 (0.984, 1.020) |

**Abbreviations:** OR, odds ratio; CI, confidence interval; sPTB, spontaneous preterm birth.
Conditional logistic regression models using a time-stratified case-crossover design. Models adjusted for mean temperature and mean temperature squared. Estimates are presented per 5 µg/m^3^ increase in PM_2.5_.

| **Supplemental Table 3.** Associations of PM_2.5_ with sPTB stratified by insurance type, *GeoBirth*, Metropolitan Philadelphia (2008-2020). | | | | |
| --- | --- | --- | --- | --- |
|  | **Daily Lag** | **sPTB OR (95% CI)** | **Averaged Lag** | **sPTB OR (95% CI)** |
| Private  (n = 1,713) | Lag 0 | 0.922 (0.862, 0.987) | Lag 0 | 0.922 (0.862, 0.987) |
|  | Lag 1 | 1.057 (0.982, 1.138) | Lag 0-1 | 0.980 (0.913, 1.052) |
|  | Lag 2 | 0.992 (0.920, 1.069) | Lag 0-2 | 1.000 (0.924, 1.081) |
|  | Lag 3 | 1.026 (0.953, 1.105) | Lag 0-3 | 1.021 (0.936, 1.112) |
|  | Lag 4 | 1.024 (0.950, 1.104) | Lag 0-4 | 1.033 (0.941, 1.134) |
|  | Lag 5 | 0.982 (0.911, 1.059) | Lag 0-5 | 1.029 (0.931, 1.138) |
|  | Lag 6 | 0.989 (0.926, 1.055) | Lag 0-6 | 1.023 (0.919, 1.139) |
| Public  (n = 2,573) | Lag 0 | 0.985 (0.931, 1.042) | Lag 0 | 0.985 (0.931, 1.042) |
|  | Lag 1 | 1.051 (0.985, 1.121) | Lag 0-1 | 1.012 (0.955, 1.072) |
|  | Lag 2 | 0.936 (0.877, 0.999) | Lag 0-2 | 0.998 (0.935, 1.066) |
|  | Lag 3 | 1.050 (0.986, 1.118) | Lag 0-3 | 1.012 (0.942, 1.087) |
|  | Lag 4 | 1.008 (0.947, 1.073) | Lag 0-4 | 1.018 (0.943, 1.099) |
|  | Lag 5 | 0.974 (0.915, 1.038) | Lag 0-5 | 1.012 (0.932, 1.100) |
|  | Lag 6 | 1.015 (0.961, 1.072) | Lag 0-6 | 1.014 (0.928, 1.109) |

**Abbreviations:** OR, odds ratio; CI, confidence interval; sPTB, spontaneous preterm birth.
Conditional logistic regression models using a time-stratified case-crossover design. Models adjusted for mean temperature and mean temperature squared. Estimates are presented per 5 µg/m^3^ increase in PM_2.5_.

| **Supplemental Table 4.** Associations of PM_2.5_ with sPTB using census tract- versus census block group-level exposure assignment, *GeoBirth*, Metropolitan Philadelphia (2008-2020). | | | | |
| --- | --- | --- | --- | --- |
|  | **Daily Lag** | **sPTB OR (95% CI)** | **Averaged Lag** | **sPTB OR (95% CI)** |
| Census tract-level | Lag 0 | 0.984 (0.952, 1.017) | Lag 0 | 0.984 (0.952, 1.017) |
|  | Lag 1 | 1.043 (1.000, 1.087) | Lag 0-1 | 1.003 (0.965, 1.042) |
|  | Lag 2 | 0.978 (0.938, 1.020) | Lag 0-2 | 1.004 (0.961, 1.048) |
|  | Lag 3 | 1.016 (0.975, 1.059) | Lag 0-3 | 1.010 (0.963, 1.059) |
|  | Lag 4 | 1.007 (0.966, 1.049) | Lag 0-4 | 1.012 (0.961, 1.066) |
|  | Lag 5 | 0.985 (0.946, 1.027) | Lag 0-5 | 1.007 (0.953, 1.065) |
|  | Lag 6 | 1.000 (0.965, 1.037) | Lag 0-6 | 1.005 (0.947, 1.067) |
| Census block group-level | Lag 0 | 0.967 (0.932, 1.004) | Lag 0 | 0.984 (0.952, 1.017) |
|  | Lag 1 | 1.042 (0.999, 1.086) | Lag 0-1 | 1.003 (0.964, 1.042) |
|  | Lag 2 | 0.979 (0.939, 1.021) | Lag 0-2 | 1.004 (0.961, 1.048) |
|  | Lag 3 | 1.016 (0.975, 1.058) | Lag 0-3 | 1.010 (0.963, 1.060) |
|  | Lag 4 | 1.007 (0.967, 1.049) | Lag 0-4 | 1.013 (0.962, 1.067) |
|  | Lag 5 | 0.986 (0.946, 1.027) | Lag 0-5 | 1.008 (0.953, 1.065) |
|  | Lag 6 | 0.999 (0.964, 1.035) | Lag 0-6 | 1.005 (0.946, 1.066) |

**Abbreviations:** OR, odds ratio; CI, confidence interval; sPTB, spontaneous preterm birth.
Conditional logistic regression models using a time-stratified case-crossover design. Census tract-level estimates were generated by aggregating PM_2.5_ and temperature exposures from the census block group to the census tract of residence at birth; census block group-level estimates are reproduced from the primary analysis (Table 2). Models adjusted for mean temperature and mean temperature squared. Estimates are presented per 5 µg/m^3^ increase in PM_2.5_.

| **Supplemental Table 5.** Associations of PM_2.5_ with sPTB using an indicator for daily mean temperature exceeding the cohort- and season-specific 90th percentile of summer temperatures, in place of linear and quadratic temperature terms, Michigan (2003-2020) and *GeoBirth*, Metropolitan Philadelphia (2008-2020). | | | | |
| --- | --- | --- | --- | --- |
|  | **Daily Lag** | **sPTB OR (95% CI)** | **Averaged Lag** | **sPTB OR (95% CI)** |
| Michigan | Lag 0 | 0.995 (0.988, 1.002) | Lag 0 | 0.999 (0.993, 1.005) |
|  | Lag 1 | 1.007 (0.999, 1.015) | Lag 0-1 | 1.001 (0.995, 1.008) |
|  | Lag 2 | 0.995 (0.987, 1.004) | Lag 0-2 | 1.001 (0.993, 1.008) |
|  | Lag 3 | 1.004 (0.996, 1.012) | Lag 0-3 | 1.001 (0.993, 1.009) |
|  | Lag 4 | 0.999 (0.991, 1.008) | Lag 0-4 | 1.000 (0.991, 1.009) |
|  | Lag 5 | 0.993 (0.985, 1.001) | Lag 0-5 | 0.996 (0.987, 1.006) |
|  | Lag 6 | 0.999 (0.992, 1.006) | Lag 0-6 | 0.994 (0.984, 1.004) |
| *GeoBirth* | Lag 0 | 0.972 (0.937, 1.008) | Lag 0 | 0.991 (0.960, 1.023) |
|  | Lag 1 | 1.044 (1.002, 1.088) | Lag 0-1 | 1.009 (0.973, 1.047) |
|  | Lag 2 | 0.979 (0.939, 1.020) | Lag 0-2 | 1.010 (0.970, 1.053) |
|  | Lag 3 | 1.017 (0.976, 1.059) | Lag 0-3 | 1.016 (0.972, 1.063) |
|  | Lag 4 | 1.007 (0.967, 1.049) | Lag 0-4 | 1.019 (0.970, 1.070) |
|  | Lag 5 | 0.986 (0.946, 1.028) | Lag 0-5 | 1.015 (0.962, 1.070) |
|  | Lag 6 | 1.001 (0.965, 1.037) | Lag 0-6 | 1.013 (0.956, 1.073) |

**Abbreviations:** OR, odds ratio; CI, confidence interval; sPTB, spontaneous preterm birth.
Conditional logistic regression models using a time-stratified case-crossover design. Models substituted an indicator for daily mean temperature exceeding the cohort- and season-specific 90th percentile of summer temperatures for the linear and quadratic mean-temperature terms used in the primary analysis. Estimates are presented per 5 µg/m^3^ increase in PM_2.5_.

| **Supplemental Table 6.** Associations of PM_2.5_ with any preterm birth (spontaneous and medically indicated combined), Michigan (2003-2020) and *GeoBirth*, Metropolitan Philadelphia (2008-2020). | | | | |
| --- | --- | --- | --- | --- |
|  | **Daily Lag** | **PTB OR (95% CI)** | **Averaged Lag** | **PTB OR (95% CI)** |
| Michigan  (n = 149,441) | Lag 0 | 0.993 (0.987, 0.999) | Lag 0 | 0.997 (0.991, 1.002) |
|  | Lag 1 | 1.009 (1.002, 1.016) | Lag 0-1 | 0.999 (0.993, 1.005) |
|  | Lag 2 | 0.991 (0.984, 0.998) | Lag 0-2 | 0.997 (0.990, 1.004) |
|  | Lag 3 | 1.007 (1.000, 1.014) | Lag 0-3 | 0.998 (0.990, 1.006) |
|  | Lag 4 | 0.996 (0.989, 1.003) | Lag 0-4 | 0.997 (0.989, 1.005) |
|  | Lag 5 | 0.997 (0.990, 1.004) | Lag 0-5 | 0.995 (0.986, 1.004) |
|  | Lag 6 | 1.001 (0.995, 1.007) | Lag 0-6 | 0.995 (0.986, 1.004) |
| *GeoBirth*  (n = 10,729) | Lag 0 | 0.991 (0.964, 1.018) | Lag 0 | 1.004 (0.981, 1.028) |
|  | Lag 1 | 1.038 (1.007, 1.069) | Lag 0-1 | 1.016 (0.989, 1.045) |
|  | Lag 2 | 0.968 (0.939, 0.998) | Lag 0-2 | 1.008 (0.977, 1.040) |
|  | Lag 3 | 1.012 (0.982, 1.042) | Lag 0-3 | 1.012 (0.978, 1.047) |
|  | Lag 4 | 1.016 (0.986, 1.046) | Lag 0-4 | 1.020 (0.983, 1.058) |
|  | Lag 5 | 0.995 (0.966, 1.025) | Lag 0-5 | 1.020 (0.980, 1.062) |
|  | Lag 6 | 0.997 (0.971, 1.023) | Lag 0-6 | 1.018 (0.974, 1.063) |

**Abbreviations:** OR, odds ratio; CI, confidence interval; PTB, preterm birth.
Conditional logistic regression models using a time-stratified case-crossover design. Outcome was any preterm birth (spontaneous or medically indicated), rather than spontaneous preterm birth alone. Models adjusted for mean temperature and mean temperature squared. Estimates are presented per 5 µg/m^3^ increase in PM_2.5_.

**Supplemental Figure 1.** Flow chart of preterm birth phenotyping in the Michigan dataset.

**Supplemental Figure 2.** Flowchart creation of study cohorts using the Michigan Dataset (A) and *GeoBirth* Dataset (B).

(A).	(B).
